# Cancer risk in relatives of *BRCA1/2* pathogenic variant carriers in a large series of unselected patients with breast cancer

**DOI:** 10.20892/j.issn.2095-3941.2022.0593

**Published:** 2023-03-02

**Authors:** Jiaming Liu, Lu Yao, Jie Sun, Li Hu, Jiuan Chen, Juan Zhang, Ye Xu, Yuntao Xie

**Affiliations:** Key Laboratory of Carcinogenesis and Translational Research (Ministry of Education/Beijing), Familial & Hereditary Cancer Center, Peking University Cancer Hospital & Institute, Beijing 100142, China

**Keywords:** *BRCA1* variant, *BRCA2* variant, cancer risk in relatives, Chinese breast cancer patients, family history of cancer

## Abstract

**Objective:**

The spectrum and risk of cancer in relatives of *BRCA1/2* pathogenic variant carriers in the Chinese population have not been established.

**Methods:**

A family history of cancer in 9903 unselected breast cancer patients was retrospectively analyzed. *BRCA1/2* status was determined for all patients and relative risks (RRs) were calculated to evaluate cancer risk in relatives of the patients.

**Results:**

The incidences of breast cancer in female relatives of *BRCA1* carriers, *BRCA2* carriers, and non-carriers were 33.0%, 32.2%, and 7.7%, respectively. The corresponding incidences of ovarian cancer were 11.5%, 2.4%, and 0.5%, respectively. The incidences of pancreatic cancer in male relatives of *BRCA1* carriers, *BRCA2* carriers, and non-carriers were 1.4%, 2.7%, and 0.6%, respectively. The corresponding incidences of prostate cancer were 1.0%, 2.1%, and 0.4%, respectively. The risks of breast and ovarian cancers in female relatives of *BRCA1* and *BRCA2* carriers were significantly higher than female relatives of non-carriers (*BRCA1*: RR = 4.29, *P* < 0.001 and RR = 21.95, *P* < 0.001; *BRCA2*: RR = 4.19, *P* < 0.001 and RR = 4.65, *P* < 0.001, respectively). Additionally, higher risks of pancreatic and prostate cancers were noted in male relatives of *BRCA2* carriers than non-carriers (RR = 4.34, *P* = 0.001 and RR = 4.86, *P* = 0.001, respectively).

**Conclusions:**

Female relatives of *BRCA1* and *BRCA2* carriers are at increased risk for breast and ovarian cancers, and male relatives of *BRCA2* carriers are at increased risk for pancreatic and prostate cancers.

## Introduction

Germline pathogenic variants in *BRCA1/2* genes result in increased risks of various cancers *via* an autosomal dominant pattern of inheritance^1^. A series of studies on the spectrum and risk of cancer in *BRCA1/2* pathogenic variant carriers (hereafter referred to *BRCA1/2* carriers) have been conducted^2–18^. It is well-known that *BRCA1* and *BRCA2* carriers are at increased risk for breast and ovarian cancers, and *BRCA2* carriers are at increased risk for pancreatic and prostate cancers^2,6,7,9–11,16–18^. The cancer risks and spectra appear to differ between *BRCA1* and *BRCA2* carriers; specifically, the risk for ovarian cancer in *BRCA1* carriers is higher than *BRCA2* carriers^2,7,17^, and the risks for pancreatic and prostate cancers in *BRCA2* carriers may be higher than *BRCA1* carriers^7,9–11,18^. The associations between *BRCA1/2* carriers and other cancers, such as colorectal and gastric cancers, have not been established, thus warrant further investigation^3–6,11–15,17,18^.

Most previous studies involving *BRCA*-associated cancer have focused on *BRCA1/2* carriers; however, a limited number of studies have examined the cancer risk and spectrum in relatives of *BRCA1/2* carriers. One study reported that relatives of *BRCA1* and *BRCA2* carriers have an increased risk for breast and ovarian cancers, and relatives of *BRCA2* carriers also have an increased risk for pancreatic cancer^19^.

In addition, the data regarding the cancer risks in relatives of *BRCA1/2* carriers are largely based on Western populations; scant data exists that pertains to Chinese populations. Chinese individuals have significantly different cancer spectra and risks compared with Western individuals, with relatively lower risks of breast and prostate cancers and relatively higher risks of gastric, esophageal, and liver cancers^20,21^. Moreover, the spectrum of *BRCA1/2* variants in Chinese women is different from Western women, with approximately 30% of variants not present in Caucasian women^22–24^. Therefore, the cancer risks in relatives of *BRCA1/2* carriers in Chinese women might be different from Caucasian women. In the present study we aimed to clarify and characterize the spectra and risks of cancers in relatives of *BRCA1/2* carriers in a large series of unselected Chinese patients with breast cancer.

## Materials and methods

### Study population

Between October 2003 and May 2015, a total of 10,341 consecutive patients with breast cancer who were treated at the Breast Center of Peking University Cancer Hospital & Institute were enrolled in a breast cancer database. All patients were confirmed to have breast cancer based on preoperative biopsy or postoperative histologic examination. *BRCA1/2* germline variants were determined in all patients by next-generation and/or Sanger sequencing, or multiplex ligation-dependent probe amplification, as described previously^25–27^. All of the *BRCA1/2* variants have been listed in detail in previous studies^25–27^. The *BRCA1/2* status in 438 patients could not be determined due to insufficient quantity or poor quality of genomic DNA. Therefore, 9903 patients with breast cancer were ultimately enrolled in this study. Among these patients, four carried pathogenic variants in both the *BRCA1* and *BRCA2* genes, and were considered *BRCA1* carriers at the time of analysis.

Variables, including demographics, clinicopathologic characteristics, and a family history of cancers, were extracted from the breast cancer database, and uncertain family history was confirmed and supplemented through telephone calls or outpatient visits.

Written informed consent was obtained during hospitalization from all patients whose clinical information and blood samples could be used for research, including genetic testing. The study was performed in accordance with the Declaration of Helsinki. Ethical approval for this study was obtained from the Ethics Committee of Peking University Cancer Hospital & Institute (Approval No. 2011KT12).

### Definitions

*BRCA1/2* variants are considered pathogenic when classified as pathogenic or likely pathogenic. Variants with uncertain significance (VUS) or likely benign or benign are considered non-pathogenic based on current American College of Medical Genetics and Genomics guidelines. Only patients with pathogenic variants were defined as carriers; other patients were considered to be non-carriers. A family history of cancers referred to cancers that occurred in first-, second- and third-degree relatives of the participants in our study. The cancer history of the participants and further relatives were not included in the analysis. A history of cancer in any relative that corresponded to the cancer in a study participant was regarded as a positive family history. Considering data accessibility, we used the family of the participants but not individuals as the unit with which to analyze the cancer risks of relatives; thus, cancer risks in relatives were calculated based on the family history of the study participants. Of note, a very small fraction of patients may have come from the same family, and such families may have been counted more than once. For convenience, related cancers with a low incidence were merged together for the analysis. For example, lymphoma included Hodgkin’s lymphoma and non-Hodgkin’s lymphoma, and urothelial carcinoma included bladder cancer and cancers of the ureter and urethra. Furthermore, fallopian tube cancer was considered ovarian cancer.

### Statistical analysis

We analyzed all types of cancer with positive family history counts equal to or greater than five among *BRCA1/2* carriers, and those with counts less than five were summarized as “other cancers”. Continuous and categorical variables are described as the means ± standard deviations (SD) and frequencies with a percentage (%), respectively. Student’s *t*-test was performed to analyze differences in continuous variables, the Wilcoxon rank-sum test was used to analyze differences in ordered categorical variables, and the Pearson chi square test or Fisher’s exact test was used to analyze differences in unordered categorical variables. In addition, we calculated relative risks (RRs) with 95% confidence intervals (CIs) for comparison of cancer risks in relatives of *BRCA1* carriers, *BRCA2* carriers, and non-carriers. All statistical analyses were performed using StataSE 15 (StataCorp LP, College Station, TX, USA). Only two-tailed *P* values < 0.05 were considered statistically significant.

## Results

### Distributions of cancers in relatives of patients with breast cancer

There were 538 germline *BRCA1/2* pathogenic variant carriers (209 for *BRCA1* and 329 for *BRCA2*) and 9365 non-carriers in this study. The distribution of cancers in the relatives of the patients with breast cancer are presented in **Table 1**. The incidences of *BRCA1* and *BRCA2* carriers among relatives with 1, 2, and ≥3 cancer types were significantly higher than non-carriers (**Table 1**). Additionally, the proportion of *BRCA1* and *BRCA2* carriers with 1, 2, and ≥3 affected family members was significantly greater than non-carriers (**Table 1**). Differences in the number of cancer types and cancer-affected members among relatives between *BRCA1* and *BRCA2* carriers, however, were not significant (**Table 1**).

**Table 1:** Demographics of patients with breast cancer and distributions of cancers in their relatives according to *BRCA* status

Variables	*BRCA1* (*n* = 209)	*BRCA2* (*n* = 329)	Non-carriers (*n* = 9,365)	*P1* value	*P2* value	*P3* value
Gender						
Male	1 (0.5)	1 (0.3)	19 (0.2)	0.357^†^	0.499^†^	1.000^†^
Female	208 (99.5)	328 (99.7)	9,346 (99.8)			
Age, years (mean ± SD)	44.7 ± 10.1	47.8 ± 10.5	51.3 ± 11.6	**<0.001^‡^**	**<0.001^‡^**	**0.001^‡^**
Cancer types						
0	76 (36.4)	146 (44.4)	6,529 (69.7)	**<0.001^§^**	**<0.001^§^**	0.063^§^
1	87 (41.6)	124 (37.7)	2,071 (23.1)			
2	35 (16.8)	45 (13.7)	574 (6.1)			
≥3	11 (5.3)	14 (4.3)	191 (1.3)			
Affected members						
0	76 (36.4)	146 (44.4)	6,529 (69.7)	**<0.001^§^**	**<0.001^§^**	0.094^§^
1	76 (36.4)	109 (33.1)	2,071 (22.1)			
2	40 (19.1)	42 (12.8)	574 (6.1)			
≥3	17 (8.1)	32 (9.7)	191 (2.0)			

*P1*, *BRCA1 vs.* non-carriers; *P2*, *BRCA2 vs.* non-carriers; *P3*, *BRCA1 vs. BRCA2*. ^†^Data comparison between two groups using the Fisher’s exact test. ^‡^Data comparison between two groups using the Student’s *t*-test. ^§^Data comparison between two groups using the Wilcoxon rank-sum test. Data are shown as *n* (%), unless otherwise specified, which refers to patients with breast cancer.

### Cancer risks in female relatives of *BRCA1* and *BRCA2* carriers

The most common cancers in female relatives of *BRCA1* carriers were breast (33.0%), ovarian (11.5%), and liver (3.8%) cancers, followed by lung and cervical cancers (2.4% each; **Table 2**). The risks for breast and ovarian cancers in female relatives of *BRCA1* carriers were significantly higher than the risks in female relatives of non-carriers (breast cancer: RR = 4.29, 95% CI = 3.50-5.27; *P* < 0.001 and ovarian cancer: RR = 21.95, 95% = CI 13.73-35.07; *P* < 0.001). Furthermore, the risks for liver (RR = 3.73, 95% CI = 1.84-7.58; *P* = 0.002) and cervical cancers (RR = 2.99, 95% CI = 1.22-7.31; *P* = 0.030) in female relatives of *BRCA1* carriers were significantly higher than female relatives of non-carriers. The cancer risks in female relatives of *BRCA1* carriers and non-carriers were not significantly different with respect to gastric, pancreatic, and colorectal cancers (**Table 2**). The most frequent cancer sites in female relatives of *BRCA2* carriers were the breast (32.2%), lung (3.7%), and ovary (2.4%), followed by the cervix and pancreas (1.5% each; **Table 2**). The risks for breast (RR = 4.19, 95% CI = 3.53-4.98; *P* < 0.001) and ovarian cancers (RR = 4.65, 95% CI = 2.22-9.73; *P* = 0.001) in female relatives of *BRCA2* carriers were also significantly higher than female relatives of non-carriers. Moreover, the risks for lymphoma (RR = 3.16, 95% CI = 1.13-8.83; *P* = 0.046) and kidney cancer (RR = 4.07, 95% CI = 1.22-13.56; *P* = 0.046) in female relatives of *BRCA2* carriers were significantly higher than female relatives of non-carriers. The pancreatic cancer risk for female relatives of *BRCA2* carriers was marginally but not significantly higher than female relatives of non-carriers (RR = 2.54, 95% CI = 1.02-6.30; *P* = 0.055). Female relatives of *BRCA1* carriers had significantly higher risks for ovarian (RR = 4.72, 95% CI = 2.16-10.31; *P* < 0.001), liver (RR = 6.30, 95% CI = 1.35-29.36; *P* = 0.016), and any cancers (RR = 1.22, 95% CI = 1.02-1.46; *P* = 0.032) than female relatives of *BRCA2* carriers (**Table 2**). Differences in the risks for other cancers (liver, gastric, and colorectal cancers) between female relatives of *BRCA1* and *BRCA2* carriers were not significant (**Table 2**).

**Table 2:** Cancer risks in female relatives of germline *BRCA1* and *BRCA2* pathogenic variant carriers

Cancers	*BRCA1* (*n* = 209)	*BRCA2* (*n* = 329)	Non-carriers (*n* = 9,365)	RR1 (95% CI, *P* value)	RR2 (95% CI, *P* value)	RR3 (95% CI, *P* value)
Breast cancer	69 (33.0)	106 (32.2)	720 (7.7)	**4.29 (3.50-5.27, <0.001^†^)**	**4.19 (3.53-4.98, <0.001^†^)**	1.02 (0.80-1.31, 0.848^†^)
Ovarian cancer	24 (11.5)	8 (2.4)	49 (0.5)	**21.95 (13.73-35.07, <0.001^‡^)**	**4.65 (2.22-9.73, 0.001^‡^)**	**4.72 (2.16-10.31, <0.001^†^)**
Lung cancer	5 (2.4)	12 (3.7)	244 (2.6)	0.92 (0.38-2.20, 0.848^†^)	1.40 (0.79-2.47, 0.247^†^)	0.66 (0.23-1.83, 0.417^†^)
Liver cancer	8 (3.8)	2 (0.6)	96 (1.0)	**3.73 (1.84-7.58, 0.002^‡^)**	0.59 (0.15-2.40, 0.775^‡^)	**6.30 (1.35-29.36, 0.016^‡^)**
Cervical cancer	5 (2.4)	5 (1.5)	75 (0.8)	**2.99 (1.22-7.31, 0.030^‡^)**	1.90 (0.77-4.66, 0.198^‡^)	1.57 (0.46-5.37, 0.521^‡^)
Gastric cancer	3 (1.4)	4 (1.2)	113 (1.2)	1.19 (0.38-3.71, 0.742^‡^)	1.01 (0.37-2.71, 1.000^‡^)	1.18 (0.27-5.22, 1.000^‡^)
Pancreatic cancer	2 (1.0)	5 (1.5)	56 (0.6)	1.60 (0.39-6.51, 0.362^‡^)	2.54 (1.02-6.30, 0.055^‡^)	0.63 (0.12-3.22, 0.711^‡^)
Endometrial adenocarcinoma	4 (1.9)	2 (0.6)	86 (0.9)	2.08 (0.77-5.63, 0.134^‡^)	0.66 (0.16-2.68, 0.771^‡^)	3.15 (0.58-17.04, 0.214^‡^)
Lymphoma	1 (0.5)	4 (1.2)	36 (0.4)	1.24 (0.17-9.04, 0.559^‡^)	**3.16 (1.13-8.83, 0.046^‡^)**	0.39 (0.04-3.50, 0.653^‡^)
Esophageal cancer	4 (1.9)	1 (0.3)	117 (1.3)	1.53 (0.57-4.11, 0.340^‡^)	0.24 (0.03-1.74, 0.192^‡^)	6.30 (0.71-55.95, 0.078^‡^)
Colorectal cancer	2 (1.0)	2 (0.6)	174 (1.9)	0.52 (0.13-2.06, 0.596^‡^)	0.33 (0.08-1.31, 0.135^‡^)	1.57 (0.22-11.09, 0.644^‡^)
Kidney cancer	0 (0.0)	3 (0.9)	21 (0.2)	0 (-, 1.000^‡^)	**4.07 (1.22-13.56, 0.046^‡^)**	0 (-, 0.286^‡^)
Urothelial carcinoma	0 (0.0)	1 (0.3)	23 (0.3)	0 (-, 1.000^‡^)	1.24 (0.17-9.14, 0.564^‡^)	0 (-, 1.000^‡^)
Other cancers	4 (1.9)	9 (2.7)	157 (1.7)	1.14 (0.43-3.05, 0.781^‡^)	1.63 (0.84-3.17, 0.146^†^)	0.70 (0.22-2.24, 0.545^†^)
Any cancer	110 (52.6)	142 (43.2)	1,746 (18.6)	**2.82 (2.47-3.23, <0.001^†^)**	**2.32 (2.03-2.64, <0.001^†^)**	**1.22 (1.02-1.46, 0.032^†^)**

RR1, *BRCA1 vs.* non-carriers; RR2, *BRCA2 vs.* non-carriers; RR3, *BRCA1 vs. BRCA2*. ^†^Data comparison between two groups using the Pearson Chi square test. ^‡^Data comparison between two groups using the Fisher’s exact test. Data are shown as *n* (%), unless otherwise specified, which refers to the number of patients with a family history of cancers.

### Cancer risks in male relatives of *BRCA1* and *BRCA2* carriers

The most common cancers in male relatives of *BRCA1* carriers were lung (7.2%), esophageal (6.2%), and gastric cancers (4.8%), followed by liver cancer and lymphoma (2.4% each; **Table 3**). Male relatives of *BRCA1* carriers had significantly higher risks of esophageal cancer (RR = 2.33, 95% CI 1= 0.36-4.00; *P* = 0.002) and lymphoma (RR = 4.77, 95% CI = 1.92-11.86; *P* = 0.005) than male relatives of non-carriers. No significant differences in the risks of other cancers (gastric, liver, and colorectal cancers) between male relatives of *BRCA1* carriers and non-carriers were detected (**Table 3**). The most common cancers in male relatives of *BRCA2* carriers were lung (4.9%), esophageal (4.9%), and liver cancers (4.3%), followed by gastric (2.8%) and pancreatic (2.7%) cancers (**Table 3**). In contrast, male relatives of *BRCA2* carriers had significantly higher risks of pancreatic (RR = 4.34, 95% CI = 2.17-8.68; *P* = 0.001) and prostate cancers (RR = 4.86, 95% CI = 2.20-10.75; *P* = 0.001) than male relatives of non-carriers. In addition, the risks of esophageal (RR =1.82, 95% CI = 1.11-2.98; *P* = 0.017), kidney (RR = 4.22, 95% CI = 1.48-11.98; *P* = 0. 020), and breast cancers (RR = 9.49, 95% CI = 1.92-46.83; *P* = 0.028) in male relatives of *BRCA2* carriers were significantly higher than male relatives of non-carriers. Differences in cancer risks between male relatives of *BRCA2* carriers and non-carriers, however, were not significant with respect to gastric, liver, and colorectal cancers. No significant differences in any type of cancer or overall cancer risk existed between male relatives of *BRCA1* and *BRCA2* carriers.

**Table 3:** Cancer risks in male relatives of germline *BRCA1* and *BRCA2* pathogenic variant carriers

Cancers	*BRCA1* (*n* = 209)	*BRCA2* (*n* = 329)	Non-carriers (*n* = 9,365)	RR1 (95% CI, *P* value)	RR2 (95% CI, *P* value)	RR3 (95% CI, *P* value)
Lung cancer	15 (7.2)	16 (4.9)	417 (4.5)	1.61 (0.98-2.65, 0.061^†^)	1.09 (0.67-1.78, 0.723^†^)	1.48 (0.75-2.92, 0.262^†^)
Esophageal cancer	13 (6.2)	16 (4.9)	250 (2.7)	**2.33 (1.36-4.00, 0.002^†^)**	**1.82 (1.11-2.98, 0.017^†^)**	1.28 (0.63-2.60, 0.497^†^)
Gastric cancer	10 (4.8)	10 (3.0)	262 (2.8)	1.71 (0.92-3.17, 0.087^†^)	1.09 (0.58-2.02, 0.794^†^)	1.57 (0.67-3.72, 0.297^†^)
Liver cancer	5 (2.4)	14 (4.3)	244 (2.6)	0.92 (0.38-2.20, 0.848^†^)	1.63 (0.96-2.77, 0.068^†^)	0.56 (0.21-1.54, 0.254^†^)
Pancreatic cancer	3 (1.4)	9 (2.7)	59 (0.6)	2.28 (0.72-7.21, 0.153^‡^)	**4.34 (2.17-8.68, 0.001^‡^)**	0.52 (0.14-1.92, 0.384^‡^)
Prostatic cancer	2 (1.0)	7 (2.1)	41 (0.4)	2.19 (0.53-8.98, 0.241^‡^)	**4.86 (2.20-10.75, 0.001^‡^)**	0.45 (0.09-2.14, 0.493^‡^)
Lymphoma	5 (2.4)	4 (1.2)	47 (0.5)	**4.77 (1.92-11.86, 0.005^‡^)**	2.42 (0.88-6.68, 0.094^‡^)	1.97 (0.53-7.24, 0.320^‡^)
Colorectal cancer	3 (1.4)	5 (1.5)	155 (1.7)	0.87 (0.28-2.70, 1.000^‡^)	0.92 (0.38-2.22, 1.000^‡^)	0.94 (0.23-3.91, 1.000^‡^)
Urothelial carcinoma	3 (1.4)	3 (0.9)	42 (0.5)	3.20 (1.00-10.24, 0.075^‡^)	2.03 (0.63-6.53, 0.196^‡^)	1.57 (0.32-7.73, 0.682^‡^)
Kidney cancer	0 (0.0)	4 (1.2)	27 (0.3)	0 (-, 1.000^‡^)	**4.22 (1.48-11.98, 0.020^‡^)**	0 (-, 0.161^‡^)
Breast cancer	1 (0.5)	2 (0.6)	6 (0.1)	7.47 (0.90-61.76, 0.143^‡^)	**9.49 (1.92-46.83, 0.028^‡^)**	0.79 (0.07-8.63, 1.000^‡^)
Other cancers	5 (2.4)	9 (2.7)	166 (1.8)	1.35 (0.56-3.25, 0.426^‡^)	1.54 (0.80-2.99, 0.197^†^)	0.87 (0.30-2.57, 0.807^†^)
Any cancer	55 (26.3)	89 (27.1)	1,562 (16.7)	**1.58 (1.25-1.99, <0.001^†^)**	**1.62 (1.35-1.95, <0.001^†^)**	0.97 (0.73-1.30, 0.851^†^)

RR1, *BRCA1 vs.* non-carriers; RR2, *BRCA2 vs.* non-carriers; RR3, *BRCA1 vs. BRCA2*. ^†^Data comparison between two groups using the Pearson Chi square test. ^‡^Data comparison between two groups using the Fisher’s exact test. Data are shown as *n* (%), unless otherwise specified, which refers to the number of patients with a family history of cancers.

## Discussion

This is the first study to determine the cancer risks in relatives of *BRCA1/2* carriers in the Chinese population. This study was based on a family history of cancer in 9903 unselected breast cancer patients. We found that the risks for breast and ovarian cancers in the female relatives of *BRCA1* and *BRCA2* carriers were significantly higher than non-carriers, and the risks for pancreatic and prostate cancers in male relatives of *BRCA2* carriers were also significantly increased.

In our study breast cancer was the most common cancer among female relatives of both *BRCA1* and *BRCA2* carriers. Ovarian cancer was the second and third most common cancer among female relatives of *BRCA1* (21.95-fold increased risk) and *BRCA2* carriers (4.65-fold increased risk), respectively. Additionally, our study demonstrated that relatives of *BRCA1* carriers have a significantly higher risk for ovarian cancer (RR = 4.72) than relatives of *BRCA2* carriers, suggesting that relatives of *BRCA1* carriers should be more aware of ovarian cancer than *BRCA2* carriers.

Pancreatic cancer is another common *BRCA*-associated cancer; however, multiple studies have shown that *BRCA2*, but not *BRCA1*, predisposes individuals to pancreatic cancer^3,7,11,19^. Our study findings were in agreement to these reports. Indeed, the pancreatic cancer risk was significantly increased in male relatives of *BRCA2* carriers (RR = 4.34) but not relatives of *BRCA1* carriers. Although pancreatic cancer risks were not shown to be significantly elevated in female relatives of *BRCA1* or *BRCA2* carriers, a trend toward risk for pancreatic cancer was still observed for female relatives of *BRCA2* carriers (RR 2.54, 95% CI 1.02-6.30; *P* = 0.055). Notably, an increased risk for pancreatic cancer was reported in female *BRCA2* carriers in a previous study^3^. Prostatic cancer is also associated with *BRCA*^3,6–11,17,18,28,29^. Similarly, we found a significantly increased risk for prostate cancer in male relatives of *BRCA2* carriers (RR = 4.86; *P* = 0.001), but a non-significant risk in male relatives of *BRCA1* carriers (RR = 2.19; *P* = 0.241); our finding is in agreement with previous studies^3,7,9,11,17,18^.

In addition, we also found that the risks for liver and cervical cancers in female relatives of *BRCA1* carriers, and lymphoma and kidney cancer in female relatives of *BRCA2* carriers were significantly increased compared with female relatives of non-carriers. Moreover, the risks of esophageal cancer and lymphoma were markedly elevated in male relatives of *BRCA1* carriers, and the risks of esophageal, kidney, and breast cancers in male relatives of *BRCA2* carriers were also increased. It should be noted that the sample size of relatives with these cancers was small, and further studies are needed.

The cancer spectrum of relatives of non-carriers was similar to the common cancer spectrum of the general Chinese population^20,30^, which may accurately reflect cancer risks in the Chinese population. Several common malignancies in the general population, such as gastric, colorectal, and endometrial cancers, were not significantly associated with *BRCA1* or *BRCA2* variants in our study, even though correlations between these cancers and variants have been reported^4,6,7,11,13^. Our study findings did not rule out the possibility of increased risks for these cancers in relatives of *BRCA1/2* carriers.

Several limitations should be considered when interpreting our study. Although the study involved a large cohort of unselected patients with breast cancer, the *BRCA1/2* carrier sample size was not sufficiently large, and some types of cancer were rare after stratification by gender and gene type. Familial cancers were based on a family history of cancers; recall bias was inevitable, especially for the cancer histories among second- and third-degree relatives.

## Conclusions

In summary, this study investigated the cancer spectrum and risk in relatives of *BRCA1/2* carriers in a Chinese population. Our findings indicated that female relatives of *BRCA1* and *BRCA2* carriers are at increased risks for breast and ovarian cancers. The risk for ovarian cancer in female relatives of *BRCA1* carriers is even higher than female relatives of *BRCA2* carriers. In addition, male relatives of *BRCA2* carriers have increased risks for pancreatic and prostate cancers. Our study may be useful for genetic counseling and cancer risk evaluation for relatives of *BRCA1/2* carriers in the Chinese population.
